# From Global to Local: A Multiscale Geographically Weighted Regression Analysis of Bovine Brucellosis Risk Factors

**DOI:** 10.1155/tbed/5449094

**Published:** 2026-06-02

**Authors:** Zihan Tian, Yingying Dong, Peng Yuan, Xiaozhong Wang, Ming Zhao, Jie Pei, Hongjin Zhao, Xianfang Xiao, Aizhen Guo, Yingyu Chen

**Affiliations:** ^1^ National Key Laboratory of Agricultural Microbiology, College of Veterinary Medicine, Huazhong Agricultural University, Wuhan, Hubei, China, hzau.edu.cn; ^2^ Hubei International Scientific and Technological Cooperation Base of Veterinary Epidemiology, The Cooperative Innovation Centre for Sustainable Pig Production, Wuhan, Hubei, China; ^3^ Yichang Animal Disease Prevention and Control Centre, Yichang, Hubei, China; ^4^ Hubei Centre for Animal Disease Control and Prevention, Wuhan, Hubei, China; ^5^ Shanghai Animal Disease Control Centre, Shanghai, China; ^6^ National Beef and Yak Industry Technology System, Yichang Comprehensive Experimental Station, Yichang, Hubei, China

**Keywords:** bovine brucellosis, multiscale geographically weighted regression, spatial epidemiology, zoonotic disease control

## Abstract

Bovine brucellosis remains a major zoonotic threat despite ongoing control measures. Conventional strategies often target broad administrative units, potentially overlooking local dynamics relevant to elimination in low‐prevalence settings. We conducted a township‐level spatial epidemiological study in southwestern Hubei Province, China, analyzing serological data from 63,222 cattle in 6335 herds collected in April 2024. Spatial clustering was assessed using Moran’s I, Getis‐Ord General G, and Local Moran’s I (LMi), while multiscale geographically weighted regression (MGWR) evaluated associations with six township‐level covariates: terrain flatness (plain‐to‐hill ratio [PHR]), road network density (RND), cattle density (Cden), goat density (Gden), large‐scale rearing ratio (LSR), and incoming cattle flow (ICF). A distinct high‐risk belt was identified in the southeast‐to‐east‐central region, with positive townships forming high–high clusters. PHR was a significant positive predictor, while RND was negative; MGWR highlighted localized positive effects of LSR. These findings demonstrate the importance of fine‐scale, geographically tailored interventions for brucellosis elimination.

## 1. Introduction

Bovine brucellosis, a zoonotic disease caused by *Brucella* species, continues to threaten global public health and livestock economies [1–3]. It is characterized by its chronic nature, tendency for recurrent outbreaks, and strong spatial clustering, leading to significant animal health and economic losses [4–7]. The integration of geographic information systems (GIS) and spatial epidemiology has become fundamental to veterinary disease research [8, 9]. These tools are critical for mapping disease distributions, identifying infection hotspots, and uncovering the environmental and husbandry factors that drive them—information that is essential for designing effective control programs.

While brucellosis control strategies are often planned at regional or national levels [8, 10], evidence increasingly shows that analyses conducted at a finer scale, such as township or village level, significantly improve the detection of local transmission hotspots. This granular approach allows for more sensitive surveillance and enables interventions that are precisely tailored to local conditions, a cornerstone of effective disease management [11–13].

A key limitation in many existing studies is the oversight of spatial nonstationarity—the phenomenon where the relationships between risk factors and disease incidence change from one location to another [14–16]. Conventional statistical models that assume a single, global relationship can mask this local variation, potentially leading to control strategies that are misaligned with the actual transmission dynamics on the ground.

To address this gap, our study investigates the spatial heterogeneity of bovine brucellosis at the township level in southwestern Hubei Province, China. By applying spatiotemporal clustering and multiscale geographically weighted regression (MGWR), we aim to (i) characterize the disease’s spatial distribution and clustering patterns and (ii) examine whether the associations between selected township‐level environmental and livestock‐production factors and brucellosis burden varied across space. The ultimate purpose was to inform more targeted surveillance and control strategies in the final stage of brucellosis control.

## 2. Methods

### 2.1. Study Area

**Figure 1:**
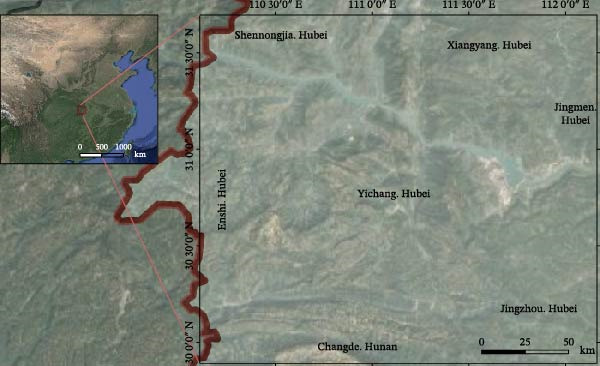
Geographic location of Yichang City with township boundaries.

This study was conducted in Yichang City, located in southwestern Hubei Province, China (29°56′–31°34′ N and 110°15′–112°04′ E). Yichang covers 21,230 km^2^ and includes multiple rural counties and extensive livestock production zones, comparable in size to a province or small state in many countries. Yichang’s geography is defined by its position on the transition boundary between China’s second and third geomorphic tiers. This location results in a highly complex topography, which we have categorized into three distinct zones: mountainous terrain in the west (~69% of the total area), hill regions in the center (21%), and eastern plains (10%) (Figure 1).

This region supports a well‐developed livestock sector and is a significant base for ruminant production within Hubei Province. This combination of diverse topography and active animal husbandry makes Yichang a critical area for brucellosis surveillance. Furthermore, the city represents a compelling case study as it has already established a brucellosis‐free zone for goats and is currently pursuing the same designation for cattle, highlighting its ongoing efforts in disease control.

### 2.2. Diagnostic Testing

Serological surveillance for bovine brucellosis in domestic ruminants followed a two‐stage serial testing algorithm, as mandated by the Chinese National Standard (GB/T 18646‐2018). All serum samples were first screened using the Rose Bengal plate test (RBPT). Samples yielding reactive results in this initial screen were then confirmed with a competitive ELISA (cELISA). Animals were definitively classified as seropositive only upon a positive result from both assays. This stringent approach prioritizes diagnostic specificity to minimize false positives.

### 2.3. Data Collection

Surveillance and farming data for 2024 were obtained from the centralized Hubei Smart Animal Husbandry and Veterinary platform to ensure temporal consistency with the serological survey. For the regression analysis, townships without cattle farming were excluded, resulting in a final sample of 93 cattle‐raising townships.

### 2.4. Spatial Clustering Analysis

Spatial clustering of bovine brucellosis at the township level was evaluated using global and local measures of spatial autocorrelation. The global pattern of clustering was assessed with Moran’s I [17], which tested for overall spatial dependence in the number of positive herds. The Getis–Ord General G statistic was then used to determine whether observed clustering was driven primarily by high or low values [18].

To identify the specific location and type of clusters, we employed Anselin’s Local Moran’s I (LMi) (local indicators of spatial association [LISA]) [19]. This analysis delineated statistically significant hotspots (high–high cluster), coldspots (low–low cluster), and spatial outliers.

A spatial weight matrix was constructed using an adaptive kernel based on the eight nearest neighbors. This approach ensures that each township has an equal number of neighbors, reducing bias introduced by variations in their sizes and shapes. The matrix was row‐standardized to interpret statistical autocorrelations as an average value of neighboring township.

The choice of eight neighbors (*k* = 8) was informed by a sensitivity analysis in which *k* values of 4, 6, 8, 10, and 12 were tested. Moran’s I remained significant across all specifications (*Z*‐scores ranging from 3.49 to 9.11). The incremental changes in *Z*‐scores were smallest between *k* = 6 and *k* = 8 (Δ*Z* = 0.796) and between *k* = 8 and *k* = 10 (Δ*Z* = 0.586), indicating that the plateau of stability lies between 6 and 10 neighbors. Therefore, *k* = 8 was selected as the minimal stable value that ensures adequate connectivity without introducing excessive spatial smoothing.

### 2.5. Covariates

Six township‐level covariates were selected based on previous spatial epidemiological studies showing that:

1. Terrain flatness (plain‐to‐hill ratio [PHR]) [9, 20]: the ratio of plain‐to‐hill area, derived from Tianditu, reflecting land surface characteristics;
2. Accessibility (road network density [RND]) [21, 22]: RND (km/km^2^), serving as a proxy for human and animal movement, obtained from the Chinese Academy of Sciences Resource and Environment Science Data Center;
3. Cattle density (Cden): The number of cattle per km^2^ (2024 data), representing the local density of susceptible bovine hosts;
4. Goat density (Gden) [23, 24]: The number of goats per km^2^ (2024 data), included as a potential factor for cross‐species exposure risk;
5. Large‐scale rearing ratio (LSR) [25]: The proportion of large‐scale cattle farms (those holding a valid Animal Epidemic Prevention Conditions Certificate) (2024 data), acting as a proxy for biosecurity and management standards;
6. Incoming cattle flow (ICF) [24]: The number of cattle introduced from other regions (2024 data), a direct proxy for external infection pressure.

All livestock data were sourced from the Hubei Smart Animal Husbandry and Veterinary platform. Prior to modeling, we assessed multicollinearity using pairwise Pearson correlations and variance inflation factors (VIFs); no covariates exhibited problematic collinearity.

### 2.6. Spatial Modeling

The outcome variable for each township was defined as the natural logarithm of the brucellosis‐positive herd count, calculated as *y*_*i*_ = log(DOT_*i*_ + 0.5), where DOT_*i*_ is the number of positive herds in township_*i*_ (a herd was classified as positive if ≥1 animal was seropositive). A conventional continuity correction of 0.5 was applied to accommodate zero counts and stabilize the variance [26].

We first fitted an ordinary least squares (OLS) regression model to establish a global baseline, estimate average effect sizes, and diagnose residual spatial autocorrelation [26]. To explicitly model the detected spatial heterogeneity, we employed MGWR with an adaptive bisquare kernel [14]. The bandwidths for the MGWR model were optimized by minimizing the corrected Akaike information criterion (AICc).

Model performance was compared using the adjusted *R* ^2^ and the AICc for small sample sizes (AICc), where a lower AICc value indicates a superior fit after penalizing for model complexity. The effectiveness of MGWR in addressing spatial nonstationarity was evaluated by testing for residual spatial autocorrelation using Moran’s I.

Finally, we mapped the local *R^2^* values and the spatially varying coefficients to visualize the geographic heterogeneity in the relationships between covariates and disease occurrence.

### 2.7. Software and Tools

All statistical and spatial analyses were conducted using the following software environments: Spatial clustering analyses, OLS regression, and mapping were performed using ArcGIS 10.0 (Esri, Redlands, CA, USA). Global and LMi calculations were conducted using the Spatial Statistics toolbox. Regression analyses (MGWR) were performed using the MGWR 2.2 software package (https://sgsup.asu.edu/sparc/mgwr).

## 3. Results

### 3.1. Seroprevalence and Descriptive Statistics

A citywide bovine brucellosis census was conducted in April 2024, testing 63,222 cattle sera from 6335 herds across Yichang. The observed individual‐level seroprevalence was 0.30% (190/63,222), and the herd‐level infection rate was 1.29% (82/6335). These low and spatially scattered prevalence rates reflect marked progress toward regional disease control targets.

### 3.2. Spatial Clustering Pattern

**Figure 2:**
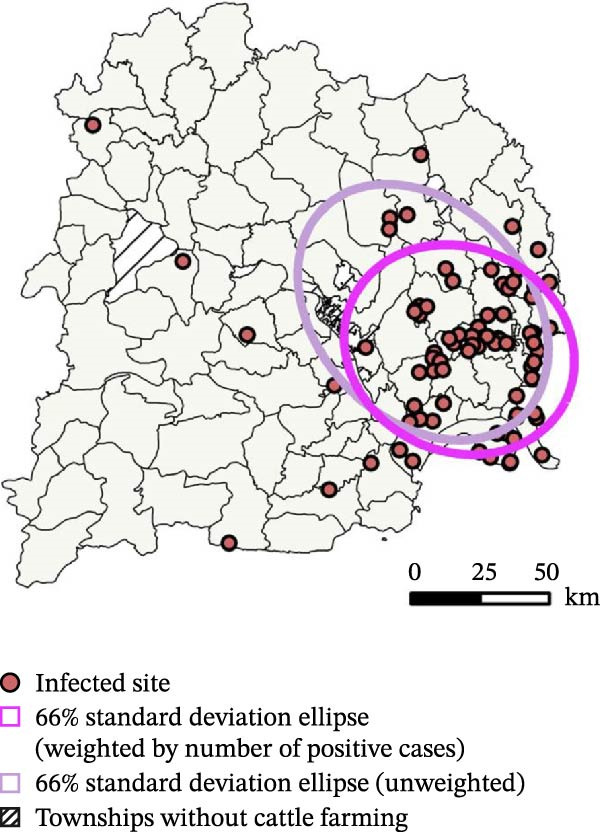
Spatial distribution of brucellosis‐positive cattle herds with 66% standard‐deviation ellipses in Yichang, China.

The spatial distribution of bovine brucellosis‐positive herds exhibited significant clustering at the township level. Analysis using standard deviation ellipses revealed a distinct pattern: An unweighted ellipse, indicating the general spread of townships reporting any infection, was oriented east–west. However, when weighed by the number of positive herds, the ellipse condensed markedly toward the southeast‐central region (semimajor axis: 44.35 km and semiminor axis: 32.85 km). This pivotal shift demonstrates that the core disease burden is concentrated in a specific southeast‐to‐east‐central belt, with only sparse reports in western and northern areas (Figure 2).

Global spatial autocorrelation analysis confirmed this nonrandom, clustered distribution. The Global Moran’s I index was statistically significant (*I* = 0.286; *Z* = 6.837; *p* < 0.001). The Getis‐Ord General G statistic further indicated that this clustering was dominated by high values (G_obs = 0.035; G_exp = 0.011; *Z* = 6.765; *p* < 0.001), confirming that townships with a high disease burden tend to be spatially adjacent.

**Figure 3:**
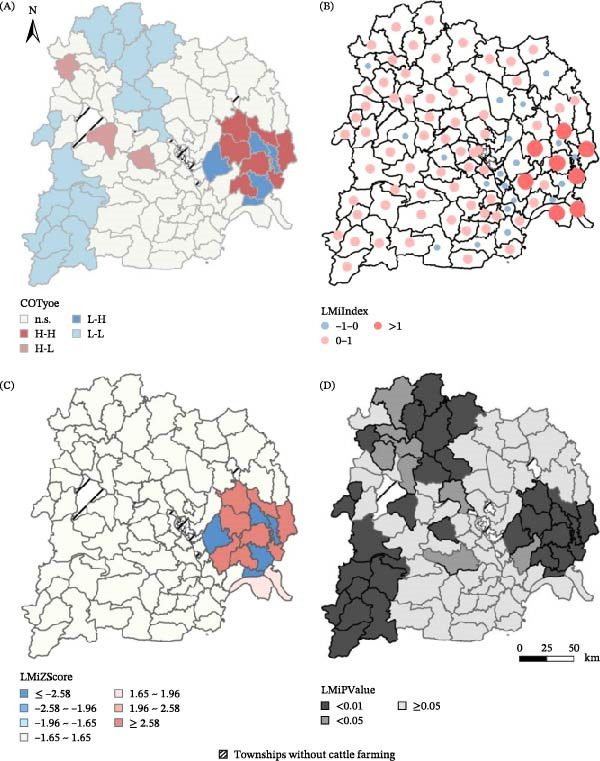
Local spatial clustering of township‐level cattle brucellosis burden in Yichang (LISA results). (A) Cluster–outlier map (LISA). (B) Local Moran’s I index (LMi). (C) Standardized LMi *Z*‐score. (D) LMi significance (*p*‐value). (LISA, local indicators of spatial association; LMi, Local Moran’s I).

Local spatial autocorrelation analysis (LISA) precisely delineated these clusters. The LISA cluster map identified high–high clusters (hotspots) in eastern Yichang and low–low clusters (coldspots) in the west (Figure 3A). High LMi values (exceeding 1.0 in several eastern townships) indicated strong local positive spatial dependence (Figure 3B). The spatial distribution of standardized LISA *Z*‐scores (LMiZScore) and their corresponding *p*‐value corroborated these findings, showing significant high–high clustering across the eastern cluster (Figure 3C,D).

Townships with no livestock were excluded from all analyses to avoid the influence of structural zeros. In summary, integrated spatial analyses consistently identified a persistent southeast‐to‐east‐central high‐risk belt for bovine brucellosis in Yichang.

### 3.3. Global Determinants: OLS Regression Results

For spatial and regression analyses, we focused on the 93 townships that maintained cattle populations. The descriptive statistics of the six township‐level covariates are summarized in Table 1. The values exhibited considerable variation, providing substantial heterogeneity for modeling. For instance, Cden ranged from 0.062 to 54.890 head/km^2^, and LSR varied from 0% to 65.587%. VIFs for all covariates were less than 5 (range: 1.096–1.700), confirming the absence of problematic multicollinearity.

**Table 1:** Descriptive statistics of covariates used in the spatial regression models (*n* = 93 townships).

Covariate	Description	Unit	Mean	SD	Min	Max	Data source
PHR	Plain‐to‐hill area ratio	%	21.897	23.283	1.328	70.010	Tianditu
RND	Road network density	km/km^2^	0.397	0.312	0.208	3.152	RESDC
Cden	Cattle density	head/km^2^	5.873	8.797	0.062	54.890	HSAHV platform
Gden	Goat density	head/km^2^	22.069	36.587	0	308.540	HSAHV platform
LSR	Proportion of large‐scale cattle farms	%	6.103	12.155	0	65.587	HSAHV platform
ICF	Inflow of cattle from other regions	head	43.398	143.437	0	1006	HSAHV platform

Abbreviations: HSAHV, Hubei Smart Animal Husbandry and Veterinary; RESDC, Resource and Environment Science and Data Center; SD, standard deviation.

An OLS regression model was fitted to establish a global baseline of the relationships between the selected covariates and log count of positive herds. The model was statistically significant (*F*‐test, *p* < 0.001) and explained ~35% of the spatial variance in township‐level incidence (adjusted *R* ^2^ = 0.354).

Diagnostic tests revealed significant spatial heterogeneity in the model residuals (Koenker BP statistic = 40.242, *p* < 0.001), indicating the presence of nonstationarity. Consequently, heteroskedasticity‐robust standard errors were used for reliable statistical inference. The residuals did not significantly deviate from normality (Jarque–Bera *p* = 0.183).

Two of the six covariates demonstrated statistically significant associations with brucellosis incidence (Table 2). RND showed a significantly negative relationship with disease occurrence (*β* = −0.270, 95% robust CI: −0.480 to −0.060, and *p* = 0.013). Conversely, the PHR exhibited a significant positive association (*β* = 0.950, 95% robust CI: 0.507–1.393, and *p* < 0.001). The remaining covariates—LSR, Cden, ICF, and Gden—did not show statistically significant associations in this global model.

**Table 2:** Global OLS model performance and heteroskedasticity‐robust coefficient estimates.

Covariates	Coefficients	Robust SE	95% robust CI	Robust_*p*
RND	−0.270	0.107	−0.480, −0.060	0.013 ^∗^
PHR	0.950	0.226	0.507, 1.393	0.000 ^∗^
LSR	0.299	0.384	−0.453, 1.051	0.438
Cden	0.003	0.007	−0.010, 0.016	0.665
Gden	−0.001	0.000	−0.001, 0.000	0.101
ICF	−0.000	0.000	−0.001, 0.000	0.658

*Note:* Model performance: adjusted *R* ^2^ = 0.354 and AICc = 234.728. Koenker (BP) test *p* = 0.000 and Jarque–Bera test *p* = 0.183.

^∗^ *p* < 0.05 (significance codes).

### 3.4. Local Heterogeneity: MGWR Model Results

To account for spatial nonstationarity observed in the OLS model, we fitted an MGWR model. Although the improvement in model fit was modest (adjusted *R* ^2^ increased from 0.354 to 0.431 and AICc decreased from 234.728 to 231.551), the MGWR provided a more appropriate representation of local heterogeneity. Importantly, the residual spatial autocorrelation observed in the global model was alleviated: the Moran’s I for OLS residuals was −0.078 (*p* = 0.090, not significant), while that for MGWR residuals was −0.098 (*p* = 0.032). The latter indicates a slight tendency toward spatial dispersion rather than clustering, consistent with MGWR capturing the underlying spatial structure and leaving only weak, nonclustered residual variation.

**Figure 4:**
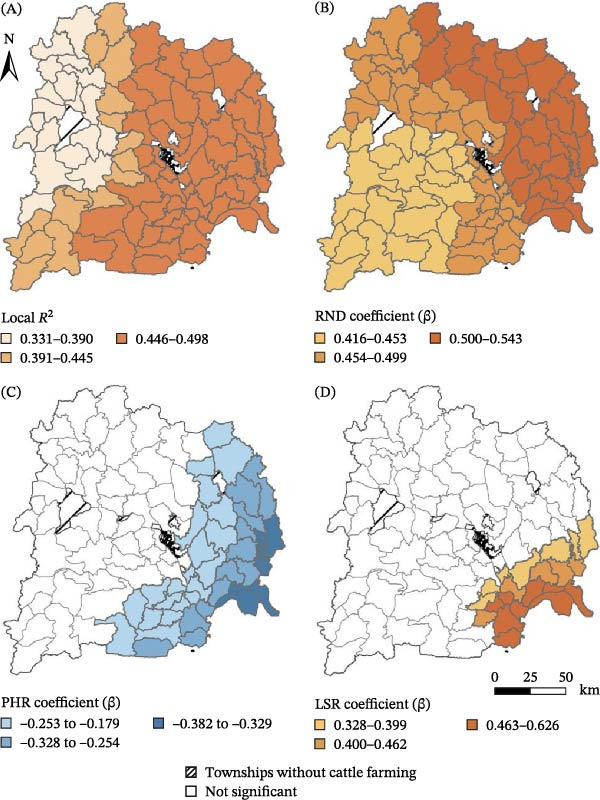
MGWR model fit and spatially varying effects on township‐level cattle brucellosis burden in Yichang, China. (A) Local model fit (local *R* ^2^). (B) Terrain flatness (plain‐to‐hill ratio [PHR]) and local coefficient (*β*). (C) Road network density (RND) and local coefficient (*β*). (D) Large‐scale rearing ratio (LSR) and local coefficient (*β*).

The spatial distribution of the local *R* ^2^ values (ranging from 0.331 to 0.498) revealed geographic disparities in the model’s explanatory power (Figure 4A). The fit was strongest in the eastern and central regions and weakest in the west, suggesting that unmeasured drivers may influence the disease risk in these areas.

The MGWR analysis refined the global results by mapping the spatial variation in individual covariate effects. PHR retained a consistently positive and stable association, with coefficients ranging from 0.416 to 0.543 across most townships (Figure 4B), pointing to its uniform role in shaping the disease risk. By contrast, the effects of RND and LSR were more localized. RND showed a band of negative coefficients (–0.382 to –0.179) concentrated in the eastern and southeastern townships (Figure 4C), while LSR displayed positive associations (0.328–0.626) in a cluster of southeastern areas (Figure 4D).

The effects of the other covariates (Cden, ICF, and Gden) were not statistically significant for the vast majority of townships, indicating their influence is likely negligible, uncertain, or too localized to be captured stably at this scale of analysis.

## 4. Discussion

Bovine brucellosis control represents a critical challenge at the human–animal–environment interface. This study conducted a fine‐scale spatial epidemiological analysis in Yichang, China, employing advanced spatial statistical methods to identify transmission patterns and drivers during the transition from disease control to elimination. The analysis yielded four main observations. First, a southeast‐to‐east‐central belt of elevated risk persisted despite the overall low prevalence. Second, RND showed a negative association with disease risk in the eastern and southeastern townships, indicating that the effect of accessibility varied across the region. Third, PHR had a positive effect across most townships, suggesting a more uniform influence. Fourth, the proportion of LSR was positively associated with risk in the southern and southeastern townships. By contrast, factors such as Cden and recorded animal trading were not significant or showed unstable effects. These findings point to the coexistence of broadly consistent and geographically specific drivers of risk and suggest that interventions need to be adapted to local conditions in the final stages of elimination.

The identification of a distinct high‐risk belt within an established control zone demonstrates that even after achieving broad‐scale success, localized transmission networks can persist. This finding aligns with global experiences in disease elimination campaigns, where residual foci often exhibit strong spatial aggregation due to localized environmental or management factors [12]. Our township‐level analysis provides the spatial precision necessary for targeted interventions, moving beyond provincial‐scale risk mappings [24] toward operational strategies that optimize resource allocation. This approach exemplifies the “precision public health” paradigm that is transforming infectious disease management worldwide.

The observed negative association between RND and brucellosis risk offers a different perspective on the role of infrastructure in disease dynamics. Although transportation networks are often implicated in facilitating the spread of infectious diseases—through increased movement of people, animals, and goods [21, 22], our results suggest that in advanced control settings, road density may instead reflect differences in service accessibility and surveillance capacity. Areas with better connectivity are more likely to benefit from timely veterinary oversight and more consistent case reporting, whereas less‐connected townships face challenges in both service delivery and detection sensitivity [27, 28]. Consequently, the higher risk observed in less‐connected areas likely reflects surveillance gaps rather than solely higher transmission intensity, necessitating active case‐finding strategies in these regions.

The stable positive association between PHR and disease risk underscores the fundamental role of environmental constraints in shaping transmission landscapes. This finding corroborates global evidence that environmental factors often provide more stable predictive signals than population metrics [9, 20]. Within the same model, Cden did not retain significance despite no problematic collinearity with terrain variables, indicating that the terrain effect is not a proxy for stocking numbers. Flatter landscapes likely prolong *Brucella* persistence due to poorer drainage and higher surface moisture, reducing natural barriers between herds, thereby sustaining higher effective contact rates even when the density is similar [29–31]. Consequently, risk‐based surveillance should treat the terrain and other environmental constraints as permanent modifiers in program planning, with particular emphasis on hygiene and segregation measures in flat areas.

In our analysis, the LSR was not a uniform driver but showed localized positive associations in the southern and southeastern townships. This pattern suggests that under certain local conditions—such as higher animal density combined with uneven implementation of biosecurity—larger operations may contribute to the sustaining risk. However, the absence of a consistent effect across the study area indicates that scale per se is not the dominant determinant [32, 33]; rather, its role depends on the regional context and management quality. This contrasts with studies emphasizing management practices over herd size [25] and points to the importance of considering both factors in tandem.

The inclusion of Gden also requires interpretation. Although Yichang has been officially recognized as a goat brucellosis‐free zone, this designation indicates that the required surveillance and control criteria have been met for a defined period rather than that all field‐level risk has been completely eliminated. In some peripheral townships, the informal exchange of breeding rams across municipal boundaries still occurs with rural areas in other cities where goat brucellosis has not reached the same low‐prevalence level. This is epidemiologically relevant because goat production in these settings relies largely on natural mating rather than artificial insemination, making breeding‐ram exchange an important link between flocks. Under such conditions, higher Gden may correspond to greater opportunities for between‐flock contact and may also reflect the possibility of cross‐species exposure at the goat–cattle interface in mixed livestock settings. For this reason, Gden was retained in the initial model as a biologically plausible indicator of the residual shared risk. At the same time, this variable should be interpreted cautiously. In the present study, Gden was not statistically significant in the global model and did not show stable local effects in most townships in the MGWR analysis, suggesting that any goat‐related influence at this scale was either limited, highly localized, or already weakened under the current control context.

The nonsignificance of official movement data reflects a structural limitation: A substantial proportion of unregistered or gray transactions bypasses regulatory oversight, creating unobserved risks that diminish the predictive value of movement‐based indicators in a disease‐free zone [24]. These hidden flows likely account for the primary threat at the current elimination stage, where the most important risks originate externally—through the introduction of live animals, contaminated semen, or feed materials. By contrast, the absence of association between regulated movements and residual foci underscores the effectiveness of official quarantine, indicating that animals passing through the formal system no longer contribute measurably to transmission during the final phase of elimination [34].

The modest improvement in model fit achieved by MGWR, while statistically significant, also indicates that conventional explanatory variables leave substantial variance unexplained in elimination settings. This suggests that, in current control settings, conventional explanatory variables may no longer capture the full set of processes shaping the residual disease risk. Similar patterns have been reported in other disease systems, where late‐stage elimination depends increasingly on finer‐scale behavioral, operational, and compliance‐related factors that are difficult to represent using routine aggregated data [35]. The residual spatial variation likely reflects unmeasured management factors including iatrogenic transmission risks [36], variations in farmer compliance, and local veterinary capacity constraints.

This study has several limitations. First, although bovine brucellosis is closely related to disease control capacity, sanitation, and farm management, we were unable to include direct township‐level indicators for these factors. Following reforms in China’s primary‐level veterinary service system, township‐level veterinary staffing and service arrangements are not readily available as stable and comparable variables for quantitative analysis, while many disease‐control measures are implemented at the county or prefecture level rather than varying systematically by township [37, 38]. For this reason, LSR was used only as a partial proxy for management and biosecurity conditions. Second, climatic factors such as temperature and humidity may influence brucellosis patterns and are therefore relevant to this question [39], but the available meteorological data were recorded at the county level or from a limited number of monitoring stations and could not be reliably assigned to individual townships without introducing substantial spatial misclassification. Third, because the analysis was conducted at the township level, some fine‐scale differences in husbandry practices, compliance, and implementation of biosecurity measures could not be captured. These constraints may partly explain the residual spatial variation observed in our models.

Taken together, these findings demonstrate that brucellosis risk in current control settings is not only shaped by the complex interaction of environmental constraints, healthcare accessibility, and management factors but also by locally variable operational conditions that are not fully visible in routine surveillance data. These findings also suggest that brucellosis control should adopt geographically stratified approaches that combine intensified surveillance in high‐risk areas with active case finding in underserved regions. Surveillance resources should be concentrated in persistent high‐risk areas, while underserved townships may require more active case finding. Routine monitoring should incorporate indicators of service accessibility and environmental risk modifiers rather than relying solely on conventional population and movement metrics. Finally, the persistence of residual foci highlights the importance of examining farm management practices and local livestock value chain networks that are not ignored in formal surveillance data.

## 5. Conclusion

In conclusion, this study found that bovine brucellosis in Yichang remained spatially clustered at the township level despite the overall low prevalence, with residual high‐risk areas concentrated in the southeast‐to‐east‐central part of the study area. It also showed that the associations between selected township‐level factors and brucellosis burden were spatially heterogeneous, with some factors showing relatively stable effects and others varying across locations. Together, these results support more geographically targeted surveillance and control and highlight the value of township‐level spatial analysis in the final stage of brucellosis control.

## Author Contributions

Conceptualization: Yingyu Chen, Aizhen Guo, Zihan Tian, and Jie Pei. Methodology: Zihan Tian. Data curation: Zihan Tian and Yingying Dong. Investigation: Zihan Tian, Yingying Dong, Peng Yuan, Xianfang Xiao, Xiaozhong Wang, and Ming Zhao. Software: Zihan Tian. Visualization: Zihan Tian. Funding acquisition: Aizhen Guo and Yingyu Chen. Project administration: Aizhen Guo and Yingyu Chen. Supervision: Xiaozhong Wang, Xianfang Xiao, and Ming Zhao. Validation: Jie Pei and Hongjin Zhao. Writing – original draft: Zihan Tian. Writing – review and editing: Yingyu Chen and Aizhen Guo.

## Funding

This work was supported by the National Key Research and Development Program of China (Grant 2025YFD1800104) and the China Agriculture Research System of MOF and MARA (Grant CARS‐37).

## Disclosure

All authors reviewed and confirmed the acceptance of the final submitted version.

## Ethics Statement

This study used secondary surveillance data with no individual animal or human subjects involved; therefore, no ethical approval was required.

## Conflicts of Interest

The authors declare no conflicts of interest.

## Data Availability

The data that support the findings of this study are available from the Hubei Provincial Department of Agriculture and Rural Affairs. Restrictions apply to the availability of these data, which were used under a license for this study. Data are available from the authors with the permission of the Hubei Provincial Department of Agriculture and Rural Affairs.
